# Weak Relationships between Stint Duration, Physical and Skilled Match Performance in Australian Football

**DOI:** 10.3389/fphys.2017.00820

**Published:** 2017-10-23

**Authors:** David M. Corbett, Alice J. Sweeting, Sam Robertson

**Affiliations:** ^1^Institute of Sport, Exercise and Active Living, Victoria University, Melbourne, VIC, Australia; ^2^Western Bulldogs Football Club, Melbourne, VIC, Australia

**Keywords:** performance analysis, sport statistics, classification tree, team sport, GPS

## Abstract

Australian Rules football comprises physical and skilled performance for more than 90 min of play. The cognitive and physiological fatigue experienced by participants during a match may reduce performance. Consequently, the length of time an athlete is on the field before being interchanged (known as a stint), is a key tactic which could maximize the skill and physical output of the Australian Rules athlete. This study developed two methods to quantify the relationship between athlete time on field, skilled and physical output. Professional male athletes (*n* = 39) from a single elite Australian Rules football club participated, with physical output quantified via player tracking systems across 22 competitive matches. Skilled output was calculated as the sum of involvements performed by each athlete, collected from a commercial statistics company. A random intercept and slope model was built to identify how a team and individuals respond to physical outputs and stint lengths. Stint duration (mins), high intensity running (speeds >14.4 km · hr^−1^) per minute, meterage per minute and very high intensity running (speeds >25 km·hr^−1^) per minute had some relationship with skilled involvements. However, none of these relationships were strong, and the direction of influence for each player was varied. Three conditional inference trees were computed to identify the extent to which combinations of physical parameters altered the anticipated skilled output of players. Meterage per minute, player, round number and duration were all related to player involvement. All methods had an average error of 10 to 11 involvements, per player per match. Therefore, other factors aside from physical parameters extracted from wearable technologies may be needed to explain skilled output within Australian Rules football matches.

## Introduction

Australian Football (AF) involves a high physical and skilled output for more than 90 min of play to maximize team performance (Gray and Jenkins, 2010). Physical and skill output may decline, as a function of time, during AF matches (Coutts et al., 2010). Consequently, a key tactical consideration during AF matches relates to the length of an on-field stint (i.e., the consecutive amount of time spent on ground by a player) for a player, before their physical and/or skilled output is adversely affected (Montgomery and Wisbey, 2016). In elite AF, there is a limitation on the number of player substitutions a team can make within a match. In the 2017 Australian Football League season, this limit was 90 rotations per match. Consequently, it is crucial in AF that stints are not ended (or started) unnecessarily early, or are too short or long in duration.

During an AF match, various athlete performance data is collected. Physical output can be measured via Global Positioning System (GPS) or Radio Frequency Identification (RFID) (Wyld, 2008; Coutts and Duffield, 2010). These devices typically sample at 10 or 15 Hz, allowing for the calculation of total distance (m), distance within velocity bands (i.e., distance covered >14.4 km·h^−1^), and peak velocity (km·h^−1^). Match statistics are provided by commercial performance analysis companies (Sullivan et al., 2014b). However, there is less standardization in the measurement of skilled output comparative to physical. Skilled output can be measured by quantifying the number of involvements or actions completed by each player. Involvements may include kicks, handballs and other actions considered important to match success by AF coaching staff. The amount of time each player spends on the field and on the bench is available as a measure of temporal output (Bradley and Noakes, 2013). Potentially due to a combination of cognitive (Tenenbaum and Bar-Eli, 1993) and physiological fatigue (Aughey, 2010), it is unlikely that players can maintain an optimal level of physical and skilled output for an entire match (Thelen and Smith, 1994; Aughey, 2010). In AF, a decrement in physical output has been observed for each quarter completed (Coutts et al., 2010), with a 3% reduction in meterage per minute for every 2 min spent on field during rotations longer than 5 min (Montgomery and Wisbey, 2016). Similarly, the level of skilled involvements for players also likely declines as the duration of a match increases. Recent research has examined how work rate, time on field and situational factors, including the number of stoppages, interact to affect skilled involvement (Sullivan et al., 2014a,b). Although factors influencing the skilled output of players have been identified to date (Sullivan et al., 2014a,b), research assessing how these factors may aid match-day stint/rotation strategies remains to be examined. Measures of skilled, physical and temporal output could be modeled to identify how the skilled output of a team and individual responds to change in temporal and physical output.

For this purpose, generalized linear mixed models present a suitable analysis option, in that they allow for the quantification of independent and dependent variables with repeated measures (Gałecki and Burzykowski, 2013). Random intercept models allow for the quantification of pooled data, whereas random slope modeling outputs differing coefficients and equations for each individual entered into the model (Eyduran et al., 2016). Consequently, the relationship between time, physical and skilled outputs at a team and individual level can be quantified.

Decision trees present an alternative, non-linear option to quantify the relationship between physical, skilled and temporal outputs. Conditional inference trees, for example, incorporate a series of significance tests to create thresholds for each dependent variable (Sardá-Espinosa et al., 2017). These thresholds create branches in the tree, each consisting of differing combinations of dependent variables, which then leads to a prediction of the independent variable. It is possible to nest participants within these trees, thus accounting for how individuals respond to differing combinations of dependent variables. This could allow examination of how physical and temporal parameters interact to influence skilled output.

Utilizing a mixed analysis approach comprised of generalized linear mixed models and conditional inference trees, this study will; (i) identify how athlete skilled output changes as a function of time in an AF match, (ii) determine the extent to which these changes occur at the individual level, and (iii) reveal how different permutations of physical and skilled parameters might correspond to differences in skilled output.

## Methods

### Participants

Professional male athletes (*n* = 39) from an elite Australian Football League (AFL) club provided written informed consent to participate in this study (age: 23 ± 4 years, height: 187 ± 8 cm, mass: 86 ± 9 kg). All participants completed at least one full match and at least one stint lasting >3 min in the 2016 AF home and away season. Ethical approval was granted by the Victoria University Human Research Ethics Committee.

### Data collection

Skilled output, defined as the sum of events completed by each player, are likely to contribute to team success as an “involvement.” This was calculated as the total of involvements completed by each player, aggregated from a timeline supplied by a commercial provider of sports statistics (Champion Data, Melbourne, Australia). Champion Data provide a timeline of key actions time stamped to each player, which can broadly be categories as; (i) disposals, (ii) other offensive actions, and (iii) defensive actions. An Excel spreadsheet was designed to aggregate the number of key involvements completed by each player within each stint. To develop the most meaningful measure of skilled output for the team included in this study, key involvements were chosen in consultation with the coaching group (Appendix 1). The sum of involvements for each player's stint was databased alongside physical data, and saved as a.csv file for analysis.

Data was collected from 14 indoor matches and 7 outdoor matches (*n* = 21) during the 2016 AFL home and away Season. For all indoor matches, athlete physical output was collected via a Catapult T5 Local Positioning System (LPS) tag (Catapult Sports, Melbourne, Australia). During outdoor matches, all participants wore a Catapult S5 GPS (Jennings et al., 2010) device (Catapult Sports, Melbourne, Australia). Both devices were worn within each player's jumpers in a custom-sewn pouch. All matches were monitored live using proprietary software Openfield (Catapult Openfield v 1.11.2-1.13.1) to ensure an adequate signal quality of >8 packets/second, and that stints were correctly recorded. At the conclusion of each match, files were synchronized to the Catapult Cloud storage system. Data for each stint was then exported into a.csv file for further analysis.

### Data cleaning

This study aimed to provide methods that were generalizable to future data. As a result, several filters were applied to the data to remove outliers (Ofoghi et al., 2013). Only stint maximum velocities in the bottom 98% of the data set (<32.2 km·h^−1^), durations in the top 95% (>3 min) and involvements in the bottom 98% (<2.2 Involvements/minute) were included in the analysis. These cut-offs were heuristically selected based on perceived practical application of the findings. All parameters were then expressed relative to stint time. Each player was assigned a random ID (1–45), whilst each stint was labeled in the format “Quarter. Stint” (i.e., the first stint of quarter 1 was labeled as 1.1). Round number was labeled from 1 to 23.

### Feature selection

Parameters included in the analyses were selected based on validity, reliability and multicollinearity features. This process was informed via a literature review on common locational parameters (Cummins et al., 2013), a correlation matrix and variance inflation matrix between all parameters. Consequently, meterage per minute (m·min-1), high intensity running (distance >14.4 km·h^−1^) per minute (m·min^−1^), very high intensity running (distance >25 km·h^−1^) per minute (VHIR·min^−1^), stint time (mins) and involvements per minute (IPM^−1^) were all selected for inclusion in the study.

### Generalized linear mixed models

Generalized linear mixed models were computed in R, using the package *lme4* (R Foundation for Statistical Computing, Vienna, Austria). For all models, player ID, stint and round number were specified as random effects, with the restricted maximal likelihood approach adopted (Gałecki and Burzykowski, 2013). A random intercept model was built to identify how skilled output changes, as a function of the other parameters, across the team. Involvements per and duration were the dependent and independent variables, respectively. Bench time, meterage per minute, high intensity running per minute and very high intensity running per minute were added to the model sequentially, with the Akaike information criteria (AIC) computed after each model to assess variable importance (Akaike, 1981). Preliminary modeling revealed that bench time (the time an athlete spent off the field between stints) had minimal impact upon model performance and it therefore was not included in the final model. Finally, a random slope model was built for each player using the remaining parameters.

### Conditional inference trees

Three conditional inference trees were constructed using the *party* package in R. This algorithm operates based on a pre-determined level of statistical significance (*p* < 0.05), and conducts recursive partitioning based on factors most strongly linked with the response variable (Sardá-Espinosa et al., 2017). For the present study, the data were split into an 80% training set and a 20% testing set. Each tree was computed with a 95% confidence interval (CI) under a Bonferroni correction and a minimum terminal node size of 100 instances. The first tree in this study utilized the same parameters as the final generalized linear mixed model. Round and stint number was removed from the second tree, whilst player ID was removed from the final tree. Each tree was cross-validated on the test data set, with model performance represented by the root mean squared error (RMSE) of involvements.

## Results

### Generalized linear mixed models

**Figure 1:**
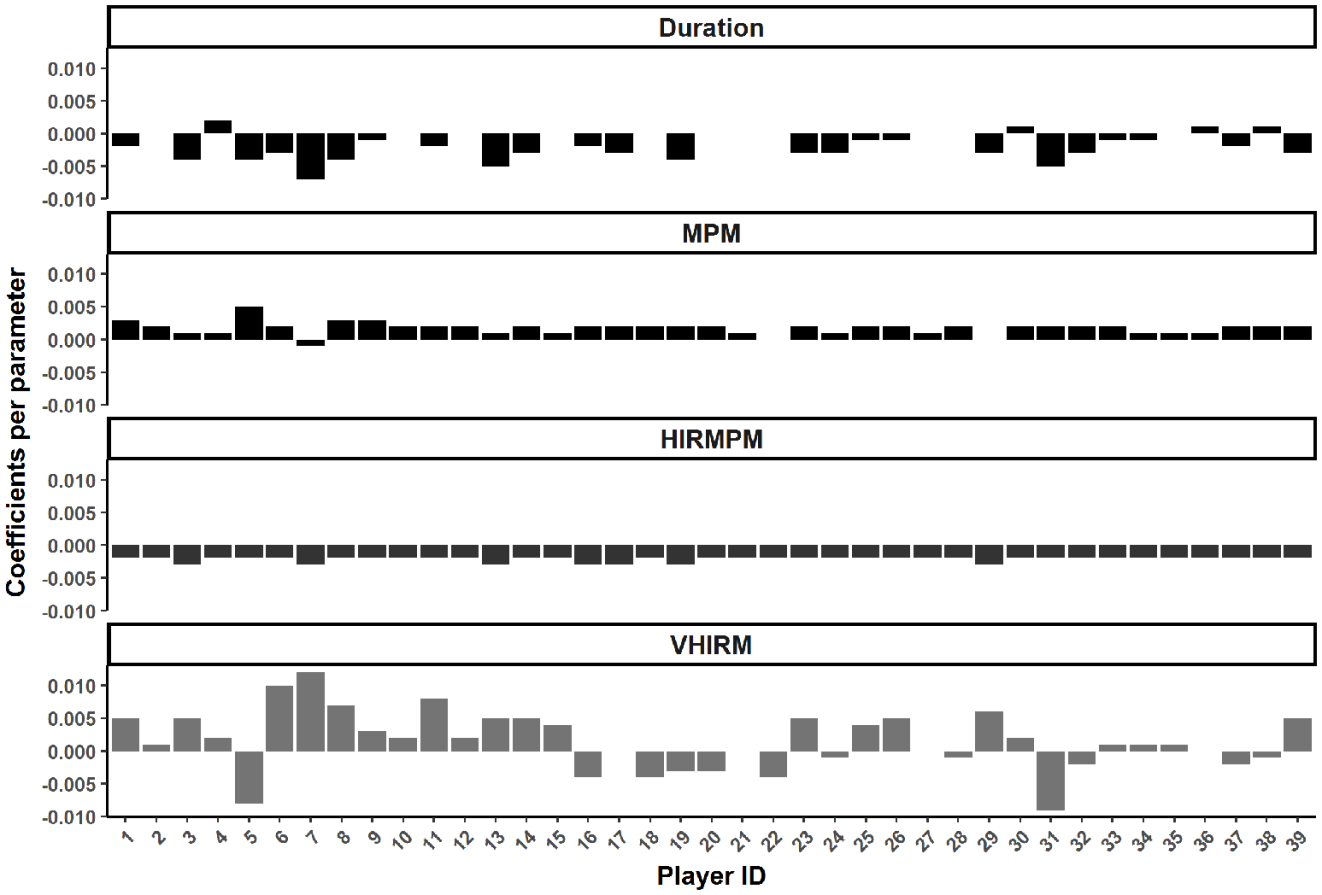
Individual coefficients for Duration (mins), meterage per minute (MPM, m·min^−1^), high intensity running per minute (HIRMPM, m·min^−1^), and very high intensity running per minute (VHIRM, m·min^−1^) in the random slope model.

Descriptive statistics of each parameter for stints (*n* = 2493) and matches (*n* = 21) are shown in Table 1. The coefficients for the random intercept model are presented in Table 2 with a 95% CI. This model had an *R*^2^-value of 0.01, and a conditional R^2^ of 0.14 (Figure 1).

**Table 1:** Descriptive statistics (mean ± SD) for; Involvements (n), duration (mins), bench time (mins), distance (m), high intensity running (HIR, distance >14.4 km·h^−1^, m), very high intensity running (VHIR, distance >25 km·h^−1^, m).

	**Stint**	**Match**
Distance (m)	1, 816 ± 903	11, 608 ± 3, 573
HIR (m)	500 ± 263	3, 198 ± 1, 165
VHIR (m)	24 ± 29	154 ± 105
Duration (mins)	13.7 ± 7.0	87.8 ± 27.2
Involvements (n)	3.6 ± 2.6	23.2 ± 9.3
Bench time (mins)	11.6 ± 9.9	74.2 ± 17.2

**Table 2:** Model 1 and 2: coefficients of fixed effects (95% confidence interval) for Intercept/Involvements per minute (IPM^−1^), Duration (mins), High intensity running per minute (HIRMPM, m·min^−1^), meterage per minute (MPM^−1^, m·min^−1^) and very high intensity running per minute (VHIRM, m·min^−1^).

	**Estimate (95% CI)**	***t*-Value**
**MODEL 1**		
Intercept (IPM^−1^)	0.108 (0.187, 0.03)	2.695
Duration (mins)	−0.001 (0, −0. 002)	−2.802
HIRMPM (m·min^−1^)	−0.002 (−0.001, −0.003)	−3.746
MPM (m·min^−1^)	0.002 (0.002, 0.001)	4.785
VHIRM (m·min^−1^)	0.003 (0.006, 0)	1.692
**MODEL 2**		
Intercept (IPM^−1^)	0.142 (0.037, 0.247)	2.648
Stint duration (mins)	−0.002 (−0. 003,0)	−2.572
HIRMPM (m·min^−1^)	0.002 (0.001, 0.003)	3.813
MPM (m·min^−1^)	−0.002 (−0.003, −0.001)	−4.490
VHIRM (m·min^−1^)	0.001 (−0.003, 0.006)	0.684

**Figure 2:**
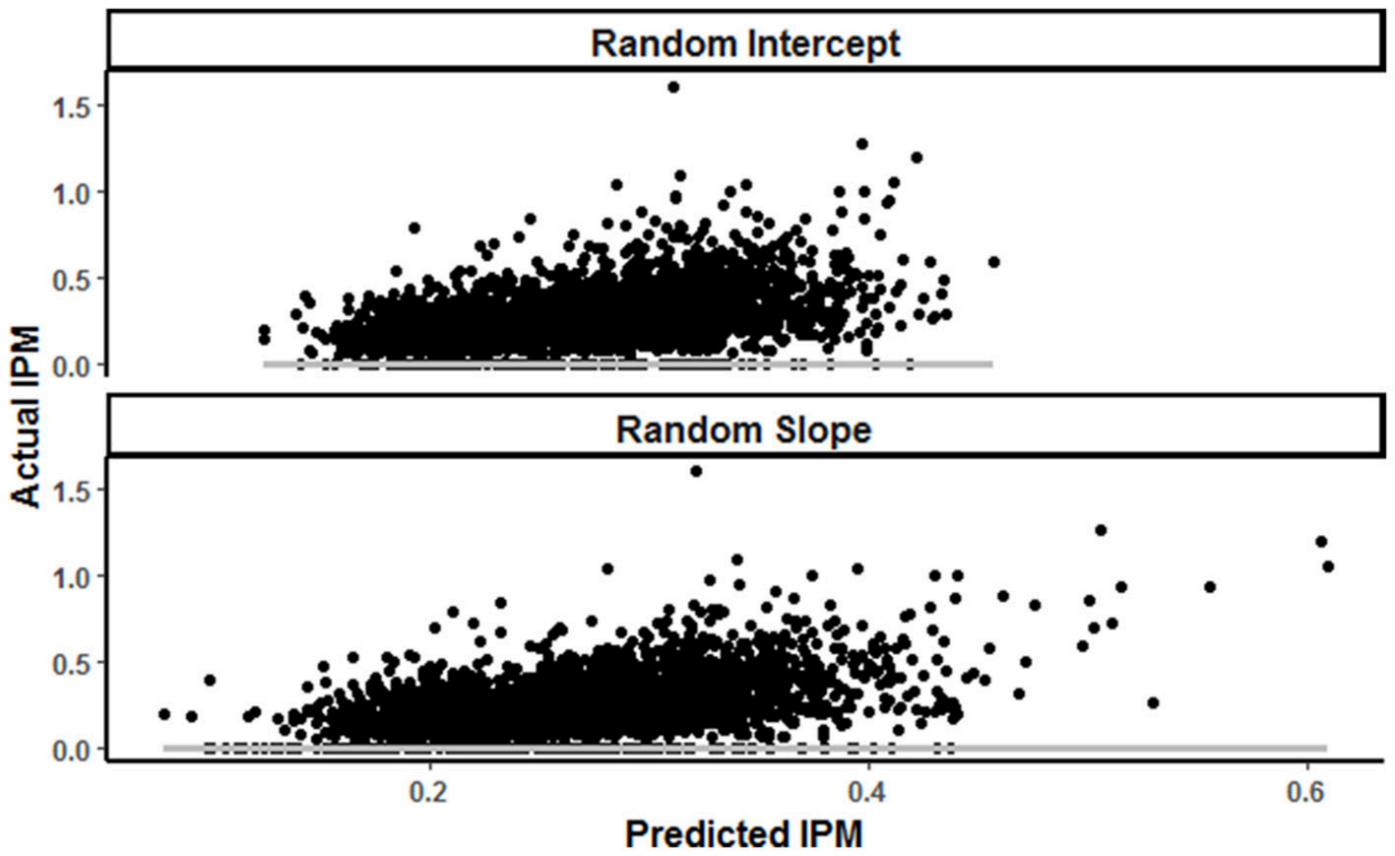
Predicted vs actual involvements per minute (IPM^−1^) in random intercept and random slope models, with gray reference line at 0 involvements of error.

The coefficients for the random slope model are presented in Figure 2. This model had an R^2^ of 0.013, and a conditional R^2^ of 0.23 (Figure 1). The relationship between both duration (for 25/39 players) and high intensity running (for 39/39 players), and involvements per minute was negative. Conversely, MPM experienced a positive relationship with involvements per minute for most players (36/39 players). The relationship between very high intensity running per minute differed considerably depending on the player. Each of these parameters had only a minor relationship with involvements, with the final model having an R^2^ of 0.012, and a conditional R^2^ of 0.23.

### Conditional inference trees

**Figure 3:**
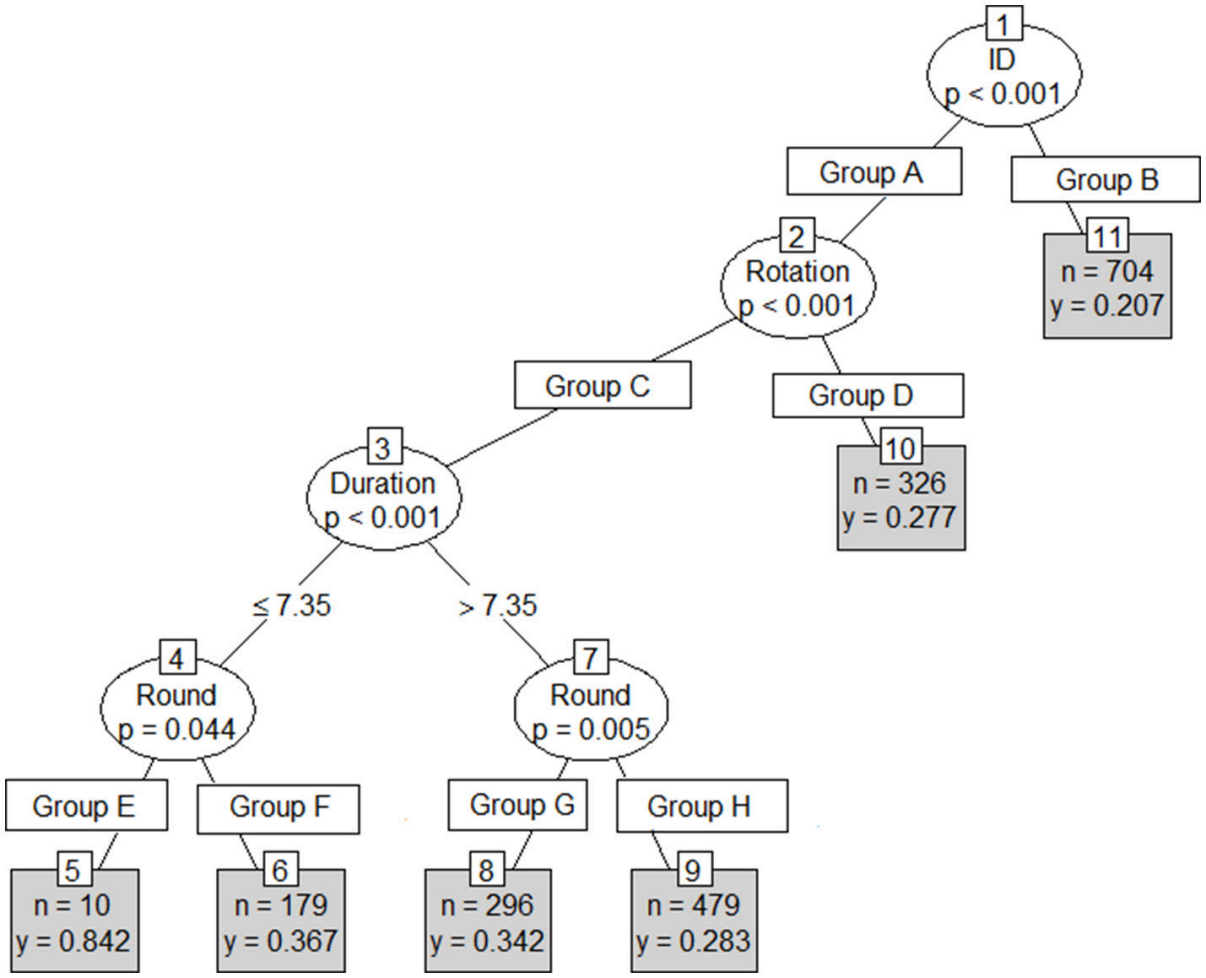
Conditional inference tree with Player ID, Round and Duration (mins) as independent variables, and involvements per minute (IPM) as the dependent variable where *n* = the number of cases in each group and *y* = predicted IPM. Group A = Player ID (1, 3, 5, 6, 7, 8, 9, 11, 13, 14, 16, 17, 19, 23, 24, 26, 29, 31, 32, 39). Group B = Player ID (2, 4, 10, 12, 15, 18, 20, 21, 22, 25, 27, 28, 30, 33, 34, 35, 36, 37, 38). Group C = Rotation (1.1, 1.2, 1.3, 2.2, 2.3, 3.1, 3.2, 3.3, 4.1). Group D = Rotation (2.1, 4.2, 4.3). Group E = Round (19). Group F: Round (1, 3, 4, 6, 7, 8, 9, 12, 13, 15, 16, 17, 18, 20, 21, 22, 23). Group G = Round (1, 2, 6, 8, 15, 17, 20, 22, 23). Group H = Round (3, 4, 7, 9, 12, 13, 16, 18, 19, 21).

**Figure 4:**
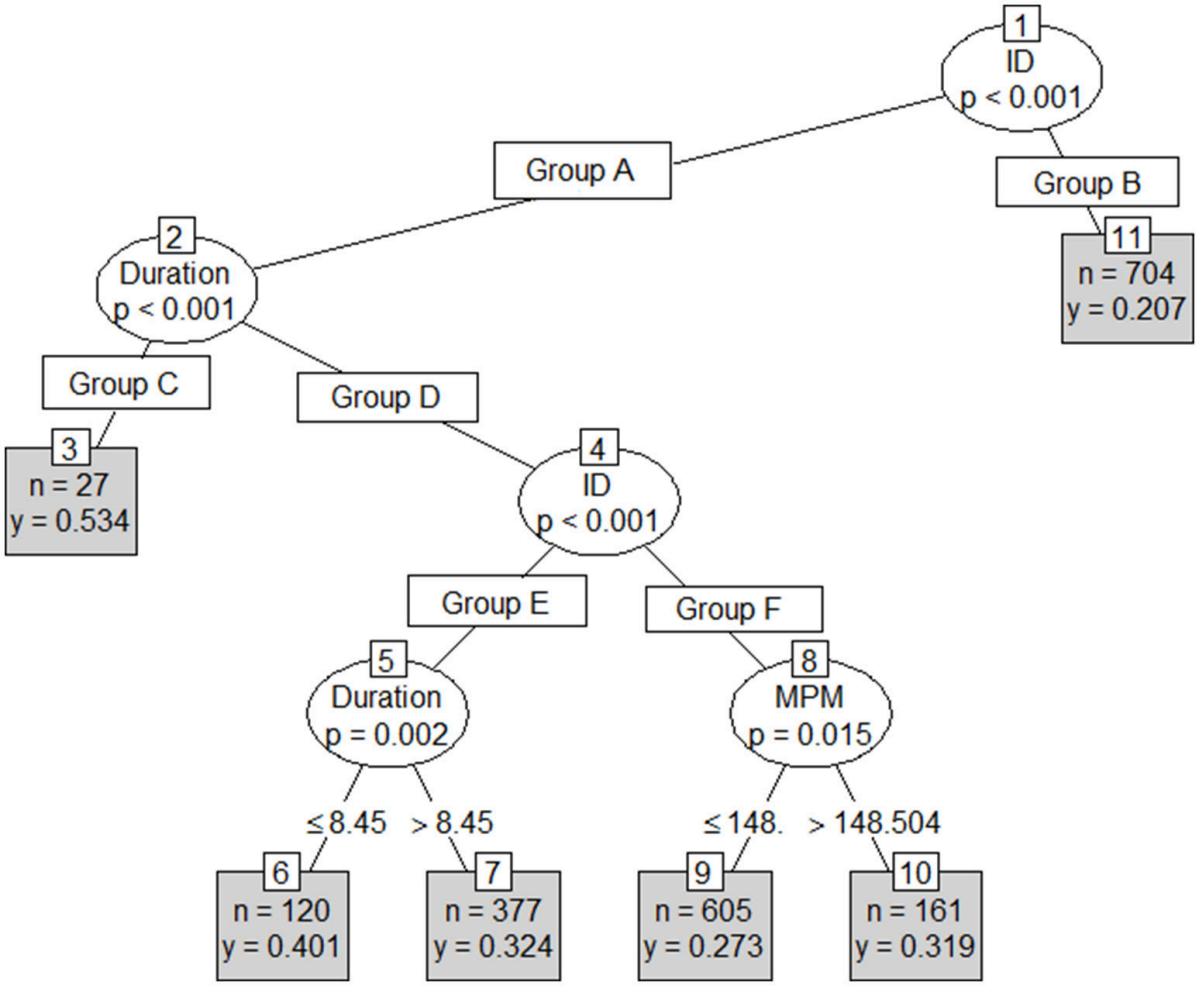
Conditional inference with Player ID, Duration (mins) and meterage per minute (MPM) as independent variables, and involvements per minute (IPM) as the dependent variable where *n* = the number of cases in each group and *y* = predicted IPM. Group A = Player ID (1, 3, 5-9, 11-17, 19, 23, 24, 26, 29, 31, 32, 39). Group B = Player ID (2, 4, 10, 12, 15, 18, 20, 21, 22, 25, 27, 28, 30, 33, 34, 35, 36, 37, 38). Group C = Duration (<5 mins). Group D = Duration (>5 mins). Group E = Player ID (3, 5, 6, 7, 8, 13, 29, 32, 39). Group F = Player ID (1, 9, 11, 14, 16, 17, 19, 23, 24, 26, 31).

**Figure 5:**
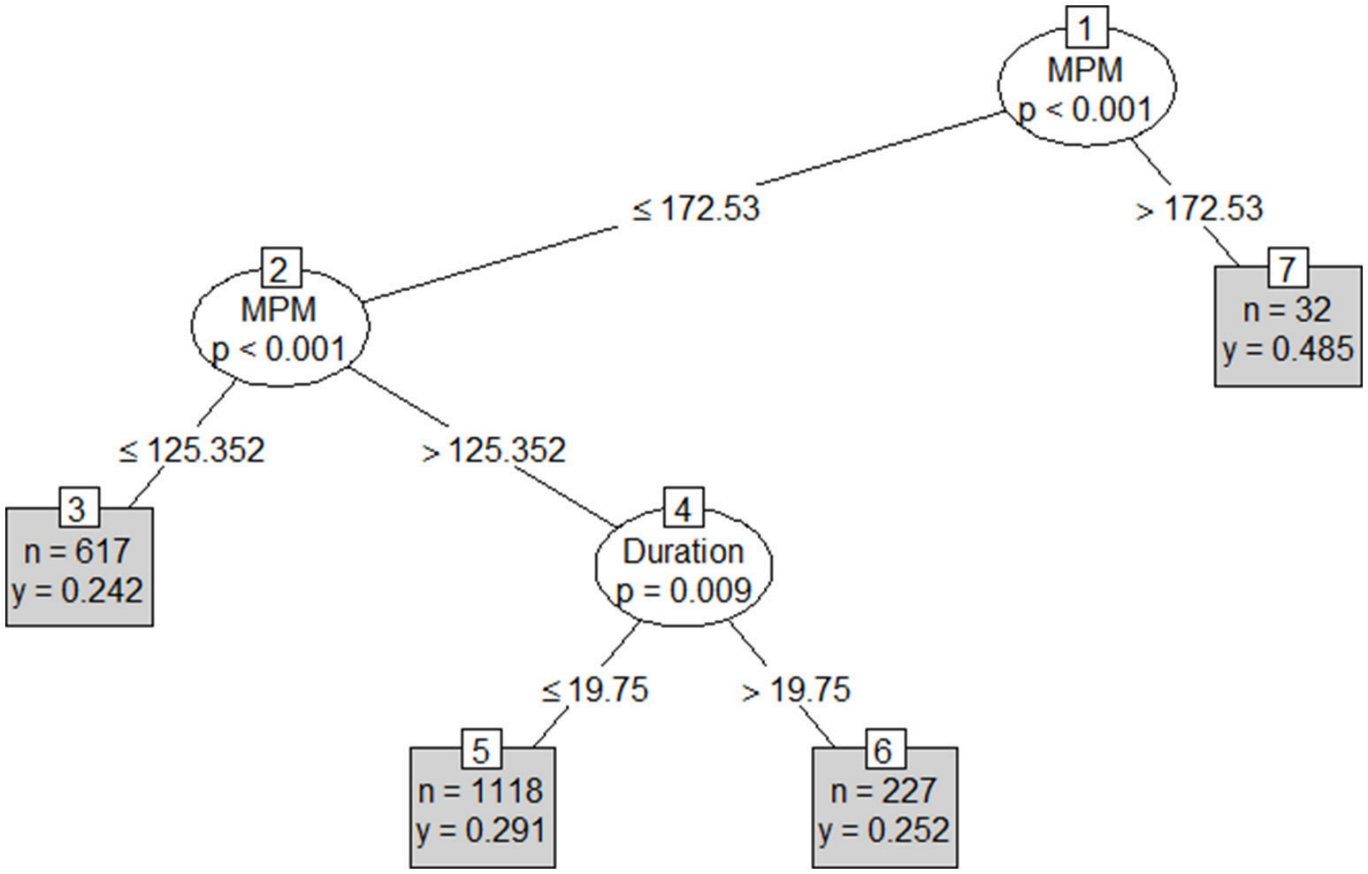
Conditional inference tree including Duration (mins) and meterage per minute (MPM) as independent variables, and involvements per minute (IPM) as the dependent variable where *n* = the number of cases in each group and *y* = predicted IPM.

Results from the first conditional inference classification tree revealed Player ID, stint number, duration and round number as the strongest indicators of involvements per minute (Figure 3). An RMSE of 0.12 involvements per minute (approximately 10.1 involvements per match) was reported on both the test and training sets. This tree's first partition included player ID, with rotation, duration and Round number forming the second to fourth partitions respectively. The second tree included player, stint duration and stint meterage per minute (Figure 4) as the strongest predictors. As per the first conditional inference tree, an RMSE of 0.12 for involvements for minute (10.1 involvements per match) was observed on both the test and train sets. This tree had an initial partition based on Player ID, with subsequent partitions based on; duration (2nd), an additional division of Player ID (3rd) and finally duration or MPM (4th). The final tree, with ID removed as an input, used only meterage per minute and stint duration to predict involvements per minute (Figure 5). An increased RMSE (0.12–0.13 involvements per minute; 11.05 involvements per match) was observed on both sets of data. In this tree, both the first and second partitions were determined using MPM, with duration only forming a partition in instances where MPM exceeded 125.

## Discussion

This study developed two methods to quantify the impact of physical outputs, on a team and individual level, on skilled output by elite AF players during matches. The first method comprised two generalized linear mixed models, resulting in broad equations for the team and individual players. Both models had low R^2^ and conditional *R*^2^-values, resulting in limited explanatory ability.

The second method, a series of conditional inference trees, identified how different circumstances and combinations of physical parameters may change an athletes' expected skilled output. Whilst partitions in the first tree were dominated by uncontrollable factors, such as round and stint number, the second tree achieved a similar classification accuracy using meterage per minute, player ID and duration. The final tree removed player ID as a parameter to identify a broad set of team rules, which only slightly reduced accuracy (0.13 compared to 0.12 involvements per minute).

The random intercept model broadly showed the strength and direction of influence for each parameter. In the observed team, meterage per minute had a negative relationship with involvements per minute. The only variable to have any positive relationship was high intensity running per minute. Practitioners could use this information as a general “rule of thumb” in match day decision making, whereby, a player who is consistently running at a high meterage per minute for an extended duration, without completing high intensity running, is less likely to reach a maximal skilled output. A limitation of this modeling technique is that it does not necessarily apply to all players, and does not identify how players individually respond to different parameters.

The random slope model addresses the above issue by allowing for different coefficients of the physical parameters for each player. This allows for better profiling of each athlete and for the importance of each parameter to better reflect an individual's strengths and weaknesses. In the observed team, for example, each of the parameters had positive and negative relationships with skilled output, depending on the player. However, despite the strengths of this modeling approach there are still limitations. The linear decline of involvements per minute declines in response to the temporal and physical inputs is assumed, when it is unlikely the decline in skilled output would be so gradual. Rather, players likely need time and physical intensity on field before their skilled output reaches an optimal level. Finally, these models suggest some level of independence between the physical and temporal parameters. As a result, they are unable to determine how parameters may interact to affect skilled output.

The first tree in this study used the same parameters entered into the random slope model, to identify how parameters interact to influence skilled output (Figure 3). However, the significance testing procedure selected uncontrollable factors, such as round and rotation numbers as the key explainers of skilled output. The first tree provided a schematic of factors that may influence skilled output in AF. However, because none of the factors from this tree are controllable within a match, this tree would likely have limited uptake in an applied setting. The second tree removed round and rotation number and partitioned based on player, stint time and meterage per minute (Figure 4). In an applied setting, the schematic created by this tree could be used to identify the conditions that are likely to lead to maximal skilled output for each player. Additionally, it could be used in a real-time monitoring setting, to identify if the current circumstances imposed upon a player are conducive to maximal skilled output.

The final conditional inference tree in this study removed player, in an attempt to generate a broad set of team rules. This could provide a cleaner schematic of influences upon skilled output across a team. Using only meterage per minute and stint time, this model set six major partitions for skilled involvement. This ranged from high physical output, but a mixed skilled output, to a low physical and low skilled output. In this playing group, a high intensity (>172 m·min^−1^), or, a moderate intensity (125–172 m·min^−1^) and moderate duration (<19.75 min) leads to a higher skilled output. Consequently, match day prescription strategies for the observed team could use this information to limit the stint time of players.

None of the models developed in this study had particularly strong accuracy. The average match duration for a player included in this study was 86 min, resulting in an average error of 0.12 IPM and equating to an average error of approximately 10.1 involvements per match. This is in agreement with other research examining the impact of contextual factors on both physical and skilled output in AF matches. In itself, physical output is influenced by factors, such as the opposition and the location of a match (Ryan et al., 2017). Furthermore, trivial relationships between common locational parameters and Champion Data player ratings as a measure of skilled performance have been noted elsewhere (Dillon et al., 2017). These findings, collectively, highlight the importance of using skilled and technical data alongside locational parameters to inform match day decision-making, as opposed to the latter alone.

There are several factors which may explain the limited relationship between GPS parameters and skilled output in AF matches. Firstly, AF is a dynamic sport, and many circumstantial details are difficult to model. In particular, opposition playing styles and changes in positions (Robertson and Joyce, 2014), may have an impact on both the physical and skilled output of player (Sullivan et al., 2014a). Secondly, the aggregate data utilized in this study is limited in its' ability to identify thresholds for reductions in both physical and skilled output. Other research has examined these outputs across quarters (Bradley and Noakes, 2013), and more recently within stints (Montgomery and Wisbey, 2016). Further work is needed to examine physical and skilled behavior as a time-series, to better describe the outputs competed by players. Finally, this was a methodological study, which aimed to identify trends across a single playing group. For this methodology to be applied to other teams and sports, the modeling approaches would need to be independently run. Therefore, the thresholds created here may not necessarily stand true outside of this playing group.

The models utilized in this study may still aid decision making in elite team sports. They use information that is controllable and readily available during matches, and therefore may assist in situations where objective information is desired to make quick, time-sensitive decisions.

## Conclusion

This study developed two methods to identify the relationship between physical, skilled and temporal outputs, on an individual and team level. The first method utilized random slope and intercept models to identify factors that may correlate with a decline in skilled output, and what direction their relationship is with skilled output. This could be used to develop a broad equation for the team and individuals, to identify how they would react to differing stint times and physical workloads. The second set of methods utilized conditional inference trees to identify how physical and temporal parameters may interact to influence skilled output. Together, these three models describe; i) the impact of uncontrollable factors, such as round and rotation number, ii) how different individuals react to different outputs and iii) a general set of thresholds for the data entered into the modeling process. These trees can provide a schematic to assist match day prescription in team sports. None of these models held an optimal predictive ability, suggesting that wearable technology data and notational analysis feeds could be analyzed differently to improve their use in team sports.

## Ethics statement

This study was carried out in accordance with the recommendations of the National Statement on Ethical Conduct in Human Research, VU Human Research Ethics Committee, with written informed consent from all subjects. All subjects gave written informed consent in accordance with the Declaration of Helsinki. The protocol was approved by the VU Human Research Ethics Committee.

## Author contributions

Data collection: DC and SR, formulation of the study: DC, AS, and SR, statistical analysis and visualization; DC and AS, first draft; DC, subsequent drafts; DC, AS, and SR, final approval; DC, AS, and SR.

### Conflict of interest statement

The authors declare that the research was conducted in the absence of any commercial or financial relationships that could be construed as a potential conflict of interest.
