# Supplementary material for: Weak Relationships between Stint Duration, Physical and Skilled Match Performance in Australian Football
Source: Front Physiol. 2017 Oct 23;8:820. doi: 10.3389/fphys.2017.00820 (PMC5660114; doi:10.3389/fphys.2017.00820)
Supplement: Supplementary file 1 [file Table1.DOCX]

**Appendix 1:** Involvements included in this study

| **Involvement Category** | **Reason for usage** |
| --- | --- |
| Disposals (Boundary Kick Ineffective, Kick In Short, Handball Effective, Handball Ineffective, Kick Effective, Kick In Long, Handball Clanger, Handball, Boundary Kick Long, Ground Kick Ineffective, Kick Long To Advantage, Kick Ineffective, Kick Backwards, Kick Inside 50, Kick Short, Kick Long, | Measure each time the player interacted with the ball |
| Offensive Actions (Mark Contested, Knock On Effective, Centre Bounce Clearance, Gather, Ball Up Hitout To Advantage, Loose Ball Get, Mark From Opp Kick, Free For, Mark Lead, Inside 50, Mark, Mark Lead, Mark Uncontested, Mark Play On, Handball Received, Shark, Hitouts to Advantage) | Measure every action which the club deems important in contributing to a goal |
| Defensive Actions (Block, Smother, Smotherer After Disposal, Run Down Tackle Dispossessed, Pressure Credit, Chase, Tackle, 1-on-1 Contest Defender, Spoil Gaining, Spoil Defensive, Hold | Measure each time a player contributed to the team by potentially preventing the oppositions’ goal. |
